# A Meta-Analysis on Prehypertension and Chronic Kidney Disease

**DOI:** 10.1371/journal.pone.0156575

**Published:** 2016-06-01

**Authors:** Yang Li, Peng Xia, Lubin Xu, Yang Wang, Limeng Chen

**Affiliations:** 1 Department of Nephrology, Peking Union Medical College Hospital, Chinese Academy of Medical Sciences and Peking Union Medical College, Beijing, China; 2 Biological Information and Statistics Center, Fuwai Hospital, National Center for Cardiovascular Disease, Chinese Academy of Medical Sciences and Peking Union Medical College, Beijing, China; Hospital Universitario de La Princesa, SPAIN

## Abstract

**Background:**

Recent studies have demonstrated that there is an association between prehypertension and an increased risk of end-stage renal disease. However, there is conflicting evidence regarding the relationship between prehypertension and chronic kidney disease (CKD). This meta-analysis aimed to demonstrate the association between prehypertension and the incidence of CKD and identify the impacts of gender and ethnic differences.

**Methods:**

MEDLINE, EMBASE, Cochrane Library (from inception through March 2016) and article reference lists were searched for relevant studies regarding blood pressure and CKD. Blood pressure (BP) measurements were classified as follows: optimal BP (less than 120/80 mmHg), prehypertension (120-139/80-89 mmHg) and hypertension (over 140/90 mmHg). CKD was defined by estimated glomerular filtration rate (eGFR)<60 ml/min/1.73 m^2^ or proteinuria. Two investigators independently extracted the data and assessed the quality of studies enrolled in this meta-analysis using the Newcastle-Ottawa Scale (NOS). We performed the meta-analysis using Stata/SE 12.0 (StataCorp LP). The random-effect models were used in the heterogeneous analyses.

**Results:**

After retrieving data from 4,537 potentially relevant articles, we identified 7 cohort studies including 261,264 subjects, according to the predefined selection criteria. Five studies were conducted in Mongolians from East Asia, and the other two studies were performed in Indo-Europeans from Austria and Iran. The participants ranged in age from 20 to 89 years, and the proportion of females ranged from 27.2% to 63.8%. The follow-up period ranged from 2 to 11 years. Compared with the optimal BP values, prehypertension showed an increased risk of CKD (pooled RR = 1.28; 95% CI = 1.13–1.44; P = 0.000; I^2^ = 77.9%). In the sex-stratified analysis, we found a similar trend in women (pooled RR = 1.29; 95% CI = 1.01–1.63; P = 0.039; I^2^ = 76.1%) but not in men. This effect was observed only in Mongolians from East Asia (pooled RR = 1.37; 95% CI = 1.18–1.59; P = 0.000; I^2^ = 81.3%) and not in Indo-Europeans.

**Conclusions:**

Prehypertension is considered a potential cause of CKD. Gender and ethnic differences are exhibited in this association.

## Introduction

The high prevalence (10%-15%) of chronic kidney disease (CKD) is now recognized as a major public health issue worldwide [1–3]. CKD is an independent risk factor of cardiovascular disease morbidity and mortality [4]. In developed countries, hypertension is the second leading cause of end-stage renal disease (ESRD), which is the last stage of CKD. Prehypertension, which was first described in the Seventh Report of the Joint National Committee on Prevention, Detection, Evaluation, and Treatment of High Blood Pressure (JNC 7) [5], has attracted increased attention from nephrologists. The relationship between prehypertension and the risk of CKD is controversial, with conflicting results derived from different cohort studies [6–12]. The Ohasama Study from Japan demonstrated a significant association between prehypertension and the development of CKD in a general population. An opposite result was observed in Iranian adults after a 10-year follow-up [6, 9]. The differences in the results of these studies may be attributed to the different cohort samples, follow-up durations, and definitions of CKD. Recently, a meta-analysis revealed that prehypertension is associated with an increased risk of ESRD [13]. To the best of our knowledge, there is no systematic review on prehypertension (120-139/80-89 mmHg) and CKD. We conducted this systematic review and prospective meta-analysis to evaluate the relationship between prehypertension and the incidence of CKD (eGFR <60 ml/min/1.73 m^2^ or proteinuria). We aimed to focus on the early diagnosis and treatment of prehypertension to prevent CKD.

## Materials and Methods

### Search Strategy

Literature searches were performed using MEDLINE, EMBASE and Cochrane Library from inception through March 2016. The search terms included the following combinations of keywords and synonyms regarding the association between prehypertension and chronic kidney disease: “blood pressure,” “prehypertension,” “pre-hypertension,” “prehypertensive,” “pre-hypertensive,” “borderline hypertension,” “high normal blood pressure,” and “chronic kidney disease,” “chronic kidney failure,” “chronic kidney insufficiency,” “chronic kidney dysfunction,” “chronic renal disease,” “chronic renal failure,” “chronic renal insufficiency,” “chronic renal dysfunction,” “end stage renal disease,” “proteinuria,” and “albuminuria.” The syntax used for PubMed is provided in Table 1, and the search strategies used for the other databases were similar, with the necessary adaptions made. There were no restrictions on language or publication forms. Additionally, we manually searched the reference lists to identify relevant studies.

**Table 1:** Search strategy for PubMed.

Step	Syntax
#1	(blood pressure[MeSH Terms]) OR prehypertension[MeSH Terms]
#2	(prehypertension[Text Word]) OR pre-hypertension[Text Word]) OR prehypertensive[Text Word]) OR pre-hypertensive[Text Word]) OR borderline hypertension[Text Word]) OR high normal blood pressure[Text Word]) OR blood pressure[Text Word]
#3	#1 OR #2
#4	(((Renal Insufficiency, Chronic[MeSH Terms]) OR Kidney Failure, Chronic[MeSH Terms]) OR proteinuria[MeSH Terms]) OR albuminuria[MeSH Terms]
#5	(chronic kidney disease[Text Word]) OR chronic kidney failure[Text Word]) OR chronic kidney insufficiency[Text Word]) OR chronic kidney dysfunction[Text Word]) OR chronic renal disease[Text Word]) OR chronic renal failure[Text Word]) OR chronic renal insufficiency[Text Word]) OR chronic renal dysfunction[Text Word]) OR end stage renal disease[Text Word]) OR proteinuria[Text Word]) OR albuminuria[Text Word]
#6	#4 OR #5
#7	#3 AND #6
#8	(risk factors[MeSH Terms]) OR risk factors[Text Word]
#9	#7 AND #8
#10	animals[MeSH Terms]
#11	humans[MeSH Terms]
#12	#10 NOT #11
#13	#9 NOT #12
#14	(epidemiologic studies[MeSH Terms]) OR cohort studies[MeSH Terms]
#15	(epidemiologic[Text Word]) OR observational[Text Word]) OR cohort[Text Word]) OR follow up[Text Word]) OR longitudinal[Text Word]) OR prospective[Text Word]) OR retrospective[Text Word]
#16	#14 OR #15
#17	#13 AND #16

### Selection Criteria

We formulated the study inclusion and exclusion criteria to collect eligible studies as previously reported [13]. We selected cohort studies that discussed prehypertension, as defined by BP values ranging from 120/80 mmHg to 139/89 mmHg in participants aged ≥18 years. BP was classified as optimal if the systolic BP was <120 mmHg and the diastolic BP was <80 mmHg, and hypertension was defined with a BP over 140/90 mmHg. The outcome was CKD or reported data eligible for CKD, as defined by an estimated glomerular filtration rate (eGFR) <60 ml/min/1.73 m^2^ or proteinuria (≥1+ using a dipstick). The estimated values of the odds ratio (OR), risk ratio (RR) or hazard ratio (HR) and its 95% confidence interval (CI) were reported. Other confounding factors, including cardiovascular risk factors such as age, sex, diabetes mellitus, body mass index and smoking, were adjusted. The following exclusion criteria were used: (1) the study reported only age- and sex-adjusted RRs without other cardiovascular risk factors; and (2) the data were extracted from the same cohort or from a secondary analysis.

### Data Extraction and Quality Assessment

Two reviewers (Yang Li and Peng Xia) independently identified potentially relevant studies by using the previously described search strategy. The titles and abstracts of each article were reviewed to ascertain the inclusion criteria, and the full text was carefully reviewed if the conformity was unclear. The data were extracted according to a standardized form, and disagreement was resolved by consensus when the data differed between the investigators. The following study characteristics were extracted: country of origin, publication year, sample size, gender, age, median follow-up time, prevalence of prehypertension, type of risk and adjusted confounding factors. We assessed the quality of the studies using the Newcastle-Ottawa Scale (NOS). In terms of study quality, the cohort studies were considered to be of fair (scores of 4–6) to good (scores of 7–9) quality.

### Data Analysis

The multivariate-adjusted outcome data (ORs, RRs, HRs and 95% CI) were transformed logarithmically in each study. The I-squared statistic was used to test for heterogeneity, and the studies were pooled using fixed effects models with low heterogeneity (I-squared <50%). Otherwise, a random-effects model was used. We assessed the publication bias using Egger’s test. The sensitivity analyses were conducted by omitting one study at a time to recalculate the pooled RR. All of the analyses were performed using Stata, version 12.0 (StataCorp LP).

## Results

### Literature Search

**Fig 1:**
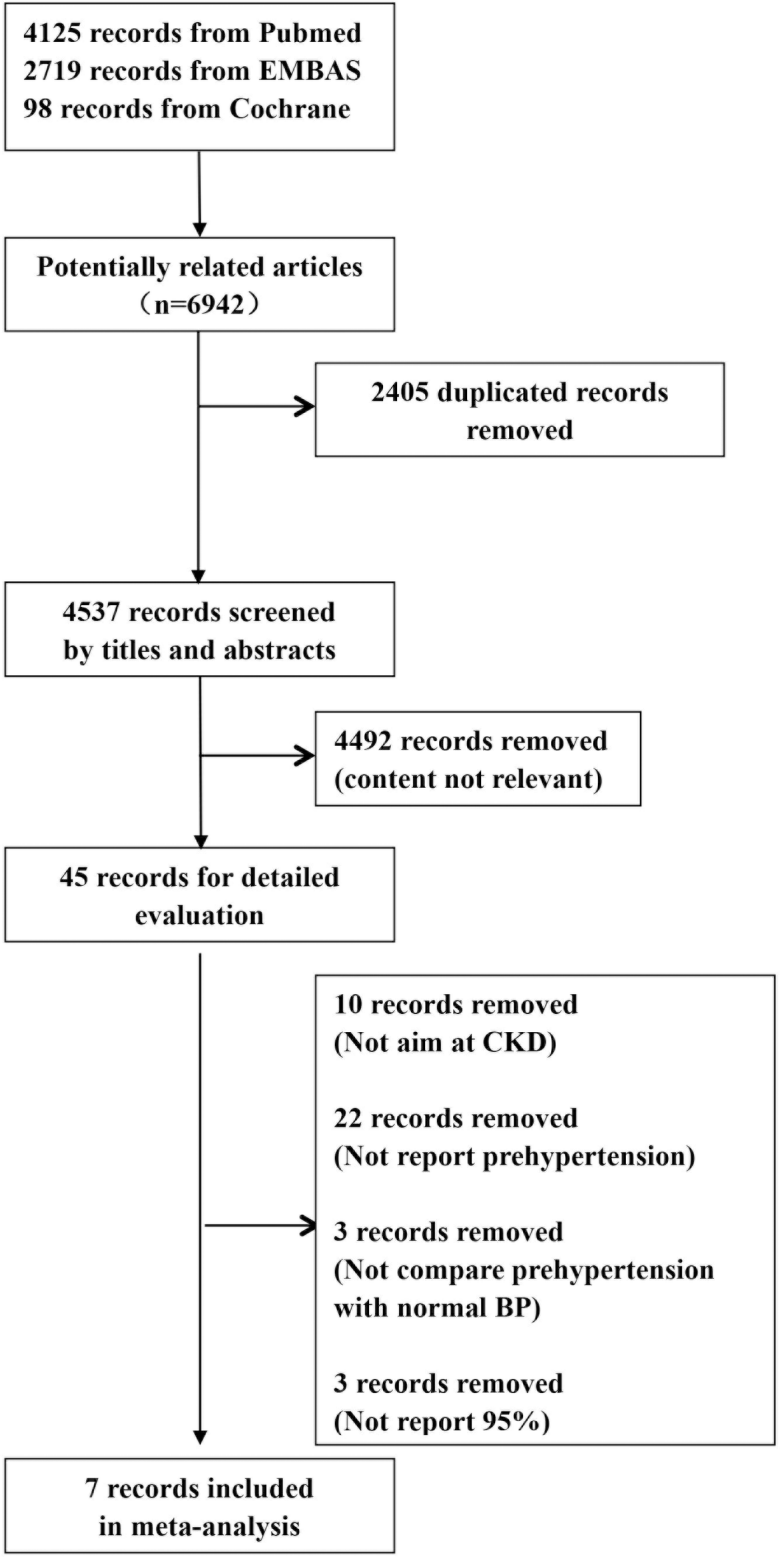
Process used for study selection. Abbreviations: BP, blood pressure; CKD, chronic kidney disease.

A total of 6,942 articles were identified using the described search strategy. Fig 1 summarizes the relevant study selection process. Approximately 2,405 duplicated records were removed. By screening the titles and abstracts, 4,492 articles were excluded because they were not relevant. After reading the full text of 45 articles, 10 articles were removed because they did not focus on CKD, 22 records were excluded because they contained no information about prehypertension, 3 were removed because they did not compare prehypertension with normal blood pressure, and the other 3 articles were excluded because they did not report 95% CI values (S1 Table). Finally, 7 studies were included in our meta-analysis [6–12].

### Study Characteristics

The baseline characteristics of the selected studies are summarized in Table 2. Five studies were based on Mongolians from East Asia, and the other two studies were based on Indo-Europeans from Austria and Iran. The population size per study ranged from 2,150 to 157,377, with a total involvement of 261,264 participants. All of the studies involved females (range, 27.2% to 63.8%), with a follow-up period ranging from 2 to 11 years. The potential confounding adjusted factors differed across studies, and the primary adjusted factors were age and sex.

**Table 2:** Baseline characteristics of the selected studies.

Study	Year	Country	Sample	Gender (Female, %)	Age (y)*	F/U (y)**	Pre-HTN Prevalence (%)*	Type of risk	Excluded Baseline CKD	Adjusted Confounding Factors
KMIC [7]	2005	Korea	157,377	33.6	35–59	10	42.63	RR	No	Age, sex, diabetes, BMI, cholesterol, smoking
VHSP [10]	2008	Austria	17,375	46.4	20–89	7 (4–11)	45.48	OR	Yes	Age, sex, BMI, smoking, uric acid, HDL, diabetes, proteinuria
Ohasama [6]	2012	Japan	2,150	63.4	60.3±9.6	6.5±4.7	37.90	OR	Yes	Age, sex, smoking, drinking, obesity, HCVD, diabetes, HTC, baseline eGFR, F/U examinations numbers
TLGS [9]	2012	Iran	3,313	56.1	≥20	9.9	Not Reported	OR	Yes	Age, sex, eGFR, diabetes, marital status, HCVD, education level, dyslipidemia, abdominal obesity, BMI, smoking, FHDM
KGES [8]	2012	Korea	6,039	52.1	52.1±8.9	2	40.72	OR	Yes	Age, sex, BMI, FBG, TC, TG, HDL-C, WC, current smoking, drinking
DCCS [11]	2014	Japan	42,625	63.77	60 (40–74)	3	58.73	OR	Yes	Age, sex, BMI
ETKC [12]	2015	China	32,385	27.2	46.40±11.57	3.9 (3.67–4.25)	61.86	HR	Yes	Age, sex, TG, LDL-C, HDL-C, FBG, smoking, drinking, diabetes

CKD was defined as an estimated glomerular filtration rate (eGFR) <60 ml/min per 1.73 m^2^ or the presence of proteinuria (1+ using a dipstick).

Pre-HTN, prehypertension; BMI, body mass index; FBG, fasting blood glucose; TC, total cholesterol; TG, triglyceride; HDL-C, high-density lipoprotein cholesterol; HTC, hypercholesterolemia; WC, waist circumference; HCVD, history of cardiovascular disease; FHDM, family history of diabetes mellitus.

*Age and Pre-HTN prevalence are baseline values.

**Values are given as the mean, mean±standard deviation, or median (range).

### Primary analysis

**Fig 2:**
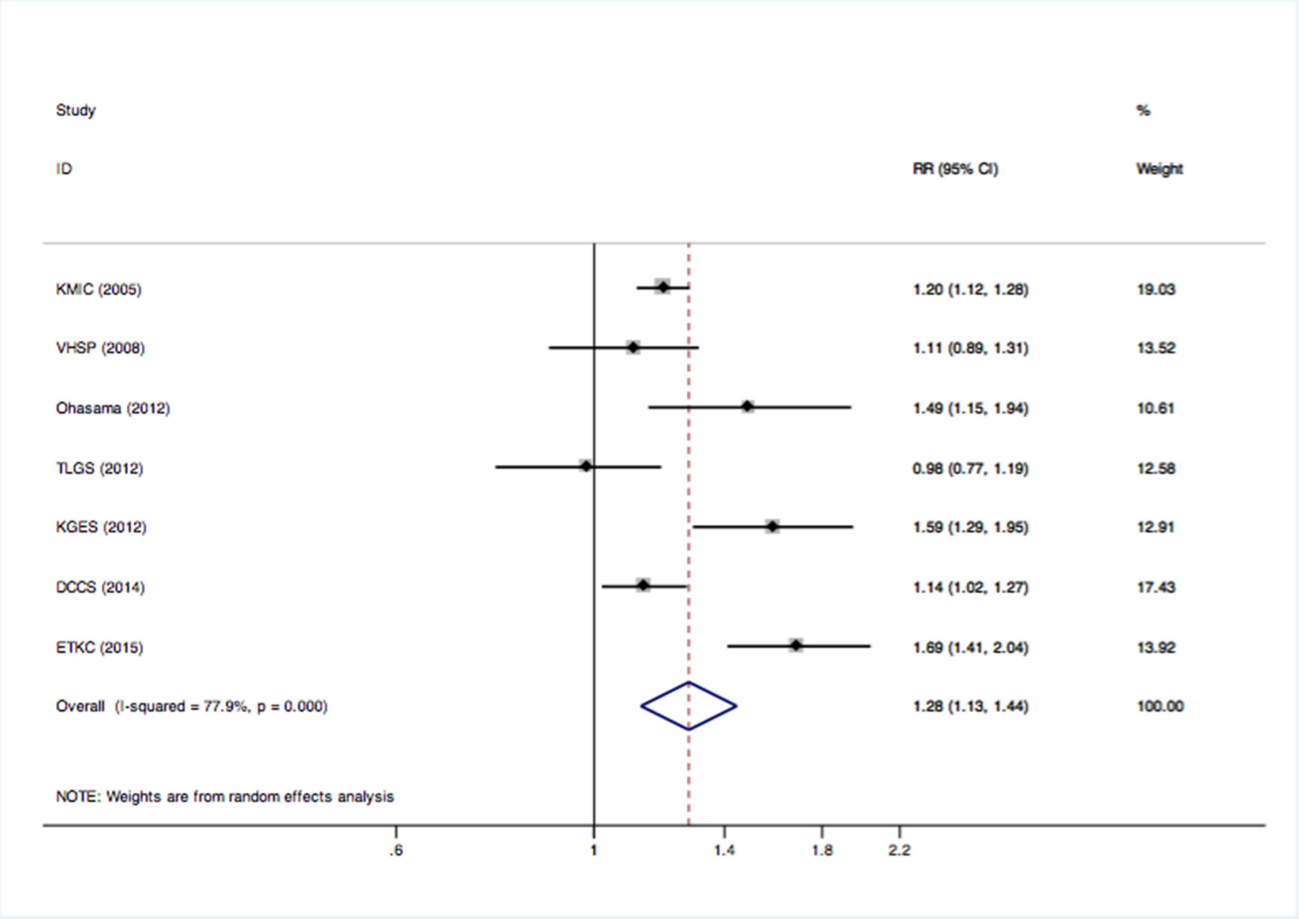
A forest plot of the association between prehypertension and CKD compared to optimal BP. **Abbreviations:** RR, risk ratio; CI, confidence interval.

The forest plot presents the association between prehypertension and CKD compared to the optimal BP (Fig 2). A statistical heterogeneity (I^2^ = 77.9%) was observed, so the random-effect model was used. The meta-analysis of the 7 studies suggested that individuals with prehypertension had an increased risk of CKD (pooled RR = 1.28; 95% CI 1.13–1.45; P = 0.000) compared to those with optimal BP values.

### Subgroup analysis

**Fig 3:**
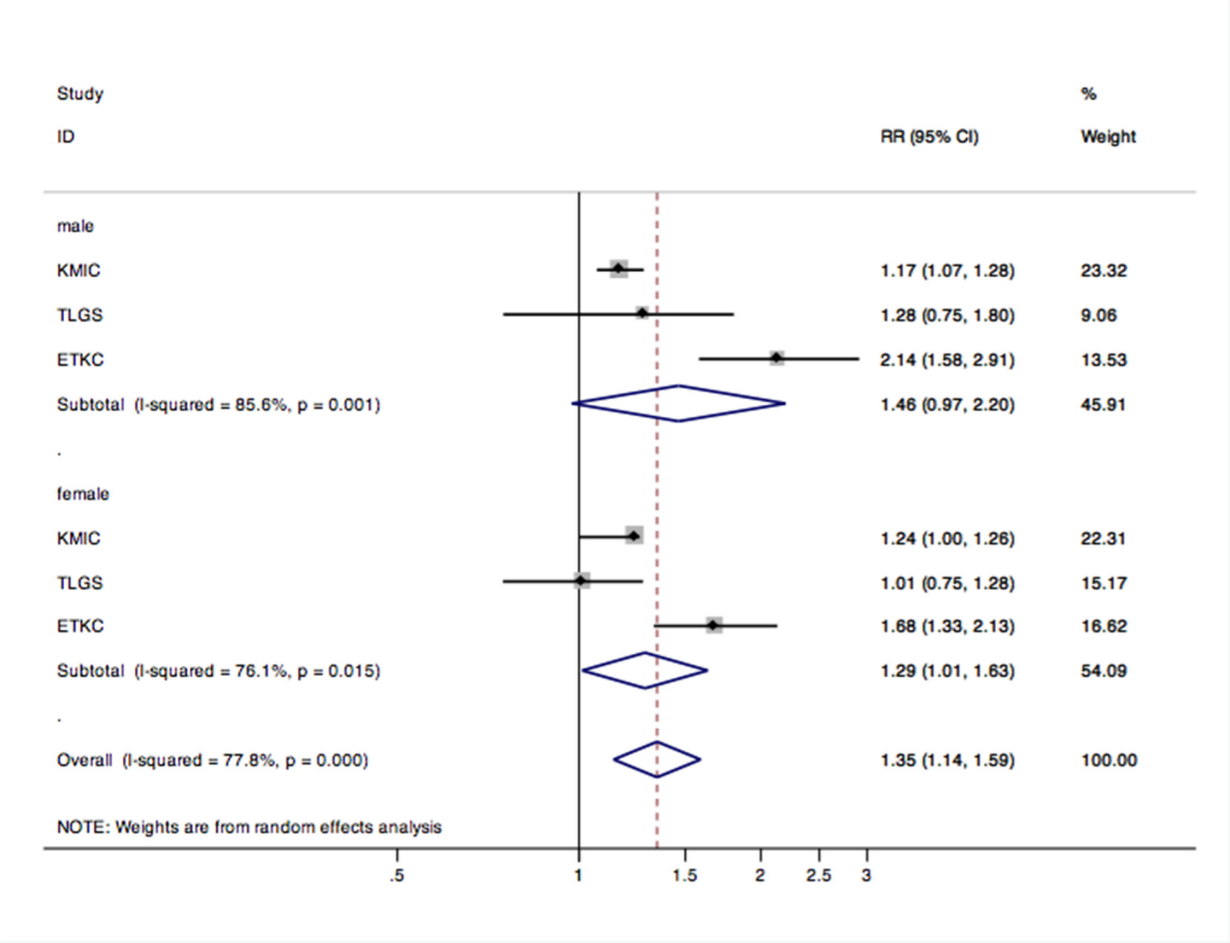
Subgroup analysis for gender difference of CKD in prehypertension compared to optimal BP. Abbreviations: RR, risk ratio; CI, confidence interval.

**Fig 4:**
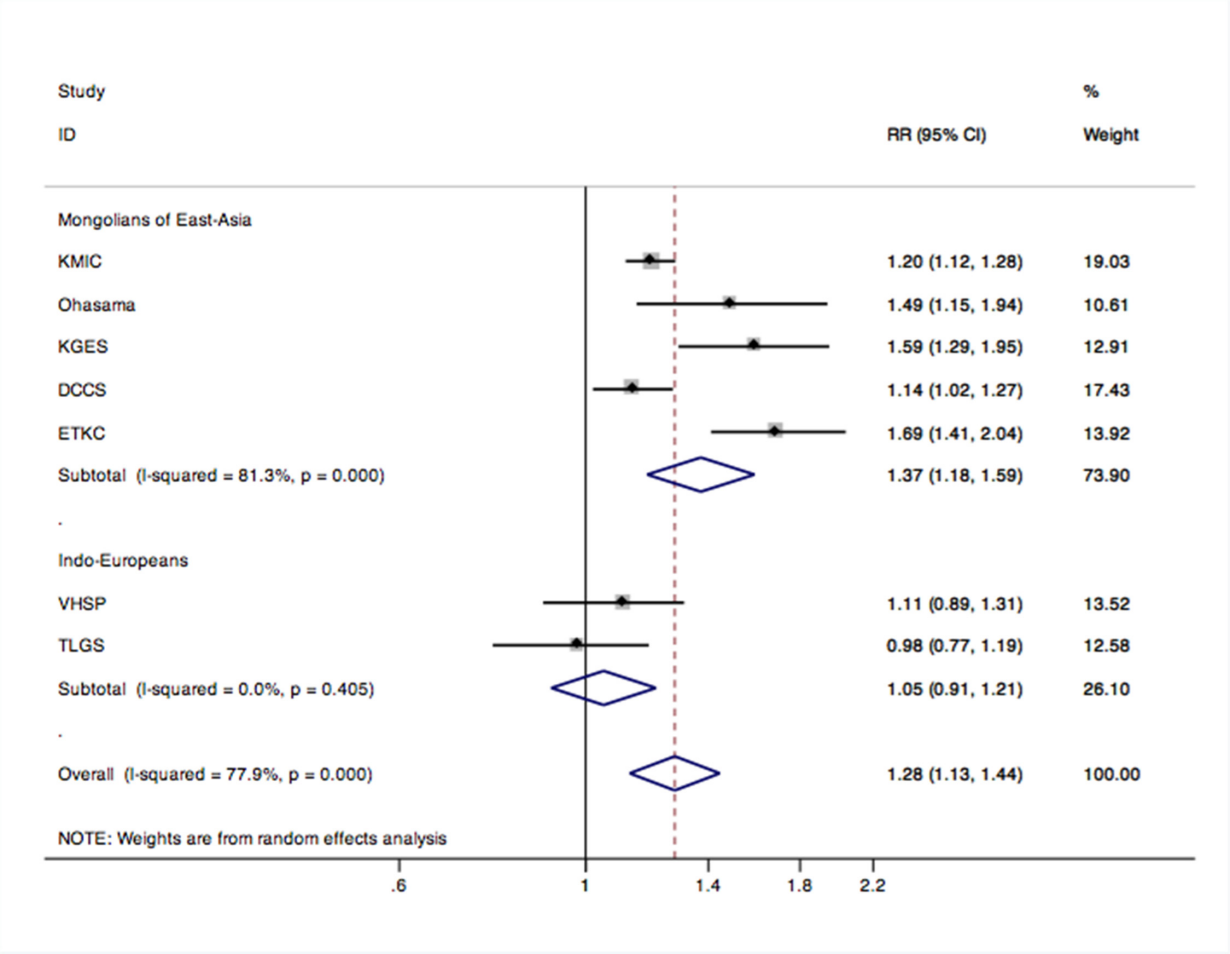
Subgroup analysis for ethnicity difference in CKD in prehypertension compared to optimal BP. Abbreviations: RR, risk ratio; CI, confidence interval.

In the subgroup analysis, we found a similar trend only in women (pooled RR = 1.29; 95% CI 1.01–1.63; P = 0.039; I^2^ = 76.1%) but not in men (pooled RR = 1.46; 95% CI 0.97–2.20; P = 0.067; I^2^ = 85.6%) (Fig 3). The effect was observed in Mongolians from East Asia (pooled RR = 1.37; 95% CI 1.18–1.59; P = 0.000; I^2^ = 81.3%) but not in Indo-Europeans (pooled RR = 1.05; 95% CI 0.91–1.21; P = 0.500; I^2^ = 0.0%) (Fig 4).

### Quality assessment

Based on the NOS quality assessment, 4 studies were defined as high quality (2 studies scored 8 and 2 studies scored 7), and the other 4 studies were defined as moderate quality (3 studies scored 6) (Table 3).

**Table 3:** Assessment of study quality.

Reference	Quality Indications of Newcastle-Ottawa Scale								Total
	1	2	3	4	5	6	7	8	
**KMIC [7]**	**Yes**	**Yes**	**Yes**	**No**	**Yes**	**Yes**	**Yes**	**No**	**6**
**VHSP [10]**	**Yes**	**Yes**	**Yes**	**No**	**Yes**	**Yes**	**Yes**	**No**	**6**
**Ohasama [6]**	**Yes**	**Yes**	**Yes**	**No**	**Yes**	**Yes**	**Yes**	**No**	**6**
**TLGS [9]**	**Yes**	**Yes**	**Yes**	**Yes**	**Yes**	**Yes**	**Yes**	**Yes**	**8**
**KGES [8]**	**Yes**	**Yes**	**Yes**	**Yes**	**Yes**	**Yes**	**No**	**Yes**	**7**
**DCCS [11]**	**Yes**	**Yes**	**Yes**	**Yes**	**Yes**	**Yes**	**Yes**	**Yes**	**8**
**ETKC [12]**	**No**	**Yes**	**Yes**	**Yes**	**Yes**	**Yes**	**Yes**	**Yes**	**7**

1. Adequate definition of Case; 2. Representativeness of cases; 3. Selection of control; 4. Definition of control; 5. Control for important factor or additional factor; 6. Exposure assessment; 7. Same method of ascertainment for cases and controls; 8. Nonresponse rate.

### Publication bias

No publication bias was identified using Egger’s test (P = 0.675).

### Sensitivity analyses

To analyze sensitivity, the primary results were not influenced by omitting one study at a time (S1 Fig).

## Discussion

In this meta-analysis including 7 cohort studies and more than 260,000 participants, we demonstrated that prehypertension is associated with a statistically significant increased risk of chronic kidney disease after controlling for several cardiovascular risk factors. The association is independent of age, sex and other risk factors, such as diabetes, body mass index (BMI), and smoking.

Prehypertension, which was called borderline hypertension or previously high-normal blood pressure, has now gained public acceptance based on the growing evidence of its association with cardiovascular diseases [14]. Prehypertension was defined by the Seventh Joint National Committee (JNC-7) in 2003 [5]. However, ways to accurately classify prehypertension and determine the need for using BP-lowering agents at this stage remained controversial. In Europe, guidelines such as the ESH-ESC Practice Guidelines for the Management of Arterial Hypertension in 2007 and 2013 were followed to maintain a high-normal BP of 130-139/85-89 mmHg [15, 16]; however, medications were not recommended. In the latest 2014 Hypertension Guideline promoted by the JNC 8, the concept was not mentioned [17]. The JNC 8 report also established higher diagnostic cut-off values for BP in several conditions, including individuals over 60 years of age and patients with diabetes and CKD. The increased diagnostic thresholds for diagnosing hypertension and ignoring prehypertension were controversial [18]. Hence, evidence regarding whether prehypertension predicts progression to hypertension and targets organ damage is important. A meta-analysis of 25 RCTs demonstrated that patients with prehypertension who received antihypertensive medications had a significant reduction in the incidence of stroke, cardiovascular events (myocardial infarction and heart failure) and mortality compared with controls [19]. Our study demonstrated meaningful evidence showing the increased risk of chronic kidney disease with elevations in blood pressure. The RR of developing CKD was 1.28 times higher in patients with prehypertension than it was in individuals with normal blood pressure, thus supporting the significance of “prehypertension” for those individuals with BP values of 120-139/80-90 mmHg.

Our findings were supported by a recent meta-analysis that included 1,003,793 participants from 6 cohort studies and concluded that the pooled RR of end-stage renal disease (ESRD) was 1.59 (95% CI 1.39–1.91) in prehypertensive subjects compared with those who had optimal BP values, after adjusting for age, sex, and ethnicity [13]. Nevertheless, there is an important difference between the studies. In our study, we broadened the analytic target to include chronic kidney disease rather than end-stage renal disease. Both eGFR <60 ml/min/1.73 m^2^ and proteinuria were used to define CKD. In fact, the prevalence of proteinuria was higher than the prevalence of having decreased eGFR among CKD patients worldwide [20, 21]. Proteinuria is also believed to be an independent risk factor of ESRD. The prevalence of CKD worldwide is over ten percent, which presents a significant challenge to the global health policy. By focusing on the potential risk factors of chronic kidney disease, we might provide new approaches for decreasing the high occurrence of this onerous global problem. In our findings, the proven association between prehypertension and CKD, rather than ESRD, enables nephrologists to focus on the early stage of chronic kidney disease, which may lead to the earlier application of interventions for prehypertension in individuals with CKD, such as lifestyle modifications or even pharmaceutical treatments [18, 22].

Recognizing that patients with prehypertension have the risk for developing full hypertension [23–25], the feasibility of using medications to prevent hypertension in patients with prehypertension was considered. Two large clinical trials showed that both candesartan and ramipril could reduce the incidence of hypertension by approximately 34% to 64% [26, 27]. Several studies regarding the clinical benefits of treating prehypertension have been conducted in the USA, Europe and China, and the outcomes included the prevention of hypertension, avoidance of target organ damage or improvement of clinical outcomes [18, 28].

In our subgroup analysis, gender and ethnic differences were observed regarding the impact of prehypertension on chronic kidney disease. Females and Mongolians from East Asia with prehypertension were prone to develop CKD, which was not mentioned in previous epidemiology studies. The clinical and public health implications of this finding warrant additional investigation. In a recent meta-analysis of more than 240,000 participants from 22 articles, the prevalence of prehypertension was higher in men than in women (41% vs. 34%) [29]. Combined with the results, it seemed that men were more likely to develop prehypertension; however, women might be more vulnerable to developing chronic kidney disease if they had previously been diagnosed as having prehypertension. Considering their risks of chronic kidney disease, special focus should be directed to females with prehypertension. Interestingly, we found that Mongolians from East Asia were more likely to suffer CKD with hypertension than Indo-Europeans were. According to previous research, in Asian-Pacific regions such as China, Japan and Korea, Mongolians from East Asia who have systemic lupus erythematosus (SLE) have shown higher rates of renal involvement compared with Indo-Europeans [30, 31]. We presumed that the condition was due to the original high prevalence of glomerulonephritis, such as lupus nephritis, in Mongolians from East Asia [31, 32]. This hypothesis implied that we should closely monitor the proteinuria and creatinine levels in Mongolians from East Asia, i.e., individuals with prehypertension from the Asian-Pacific region. Additional evidence is required to enable us to make this conclusion.

Our study has several limitations. One of the cohort studies selected in the comprehensive analysis lacked information concerning whether the participants had CKD (KMIC study) at the threshold of the original follow-up. This lack of information could confound the causality when we speculated on the association between blood pressure and CKD. Those individuals with renal insufficiency might motivate us to evaluate blood pressure measurements. Because of the various factors that impact the occurrence of CKD, there might be several potential residual confounding variables in our study, although we only included multivariate-adjusted studies to minimize the influence. The study with the largest sample size had a lower proportion of females (KMIC study), which might weaken the meaning of the subgroup analysis of gender. Because the majority of the studies included in the meta-analysis are from Asia, more data are needed to validate this relationship in other areas. Furthermore, we failed to collect sufficient data regarding the prevalence of proteinuria in individuals with prehypertension (TLGC study); thus, a more detailed relevant analysis would be limited. Additional studies aimed at evaluating the relationship between prehypertension and proteinuria might be available with more data.

In conclusion, those individuals with prehypertension defined as blood pressure of 120/80 mmHg to 139/89 mmHg have an increased risk of chronic kidney disease, which emphasizes the significance of prehypertension. This condition warrants more attention in Mongolians from East Asia and in females. Well-designed cohort studies stratified by blood pressure ranges are required in the future.

## Supporting Information

S1 Checklist PRISMA Checklist. (DOC)

S1 Fig Primary data of the sensitivity analysis by omitting one study at a time. (TIF)

S1 File PRISMA Flow Diagram. (DOC)

S1 Table Full-text excluded articles with reasons for exclusion (n = 38). (DOC)
