# Supplementary figures and images for: A Meta-Analysis on Prehypertension and Chronic Kidney Disease
Source: PLoS One. 2016 Jun 1;11(6):e0156575. doi: 10.1371/journal.pone.0156575 (PMC4889081; doi:10.1371/journal.pone.0156575)

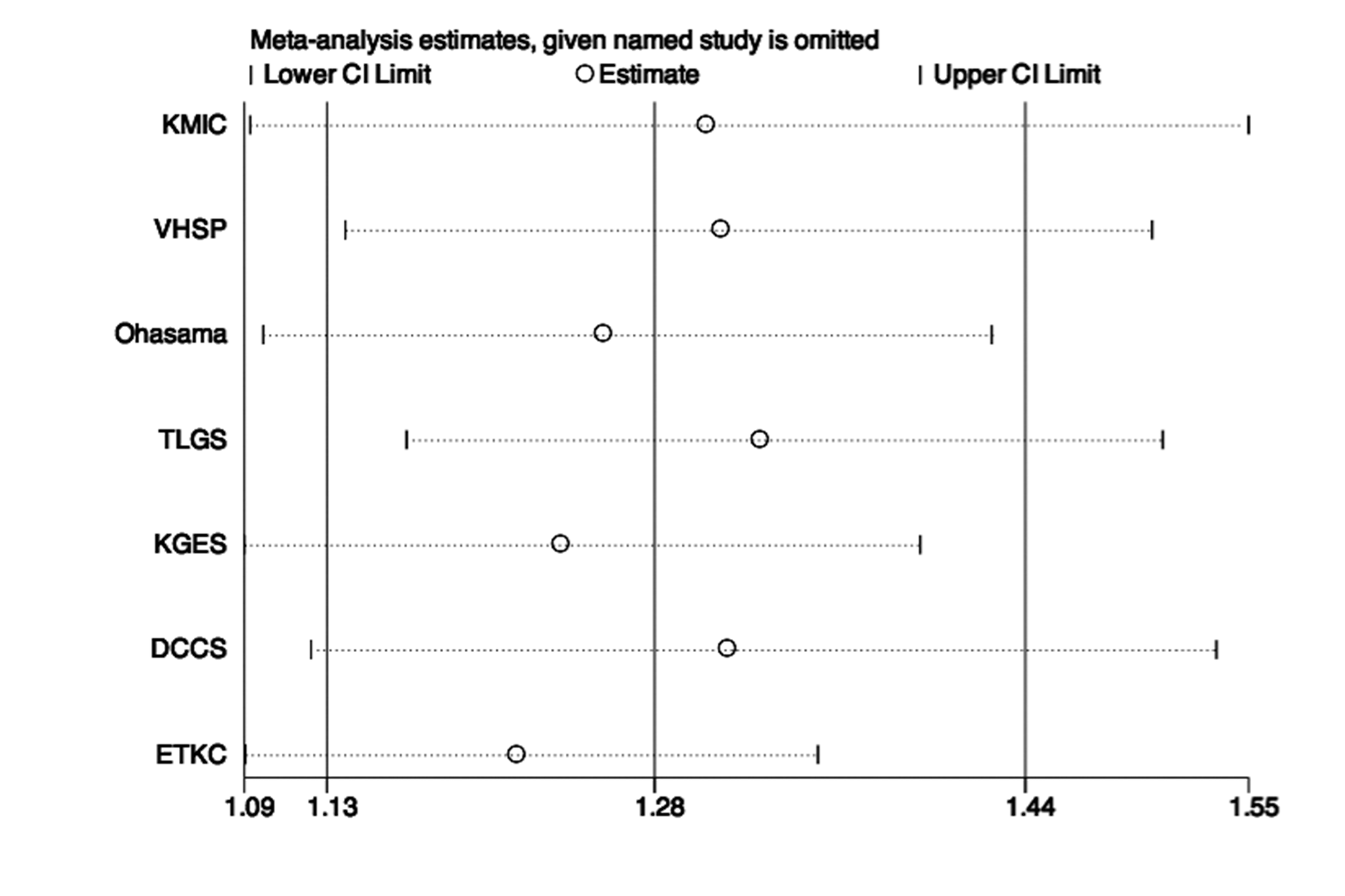

Supplement: S1 Fig — (TIF) [file pone.0156575.s002.tif]
