# Supplementary material for: A Meta-Analysis on Prehypertension and Chronic Kidney Disease
Source: PLoS One. 2016 Jun 1;11(6):e0156575. doi: 10.1371/journal.pone.0156575 (PMC4889081; doi:10.1371/journal.pone.0156575)
Supplement: S1 File — (DOC) [file pone.0156575.s003.doc]

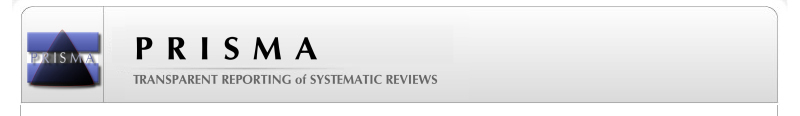
**PRISMA 2009 Flow Diagram**

**Screening**

**Included**

**Eligibility**

**Identification**

Records identified through database searching
(n = 6942 )

Additional records identified through other sources
(n = 0 )

Records after duplicates removed
(n = 4537 )

Records screened
(n = 4537 )

Records excluded
(n = 4492 )

Full-text articles assessed for eligibility
(n = 45 )

Full-text articles excluded, with reasons
(n = 0 )

Studies included in qualitative synthesis
(n = 45 )

Studies included in quantitative synthesis (meta-analysis)
(n = 7 )
