# Supplementary material for: A Meta-Analysis on Prehypertension and Chronic Kidney Disease
Source: PLoS One. 2016 Jun 1;11(6):e0156575. doi: 10.1371/journal.pone.0156575 (PMC4889081; doi:10.1371/journal.pone.0156575)
Supplement: S1 Table — (DOC) [file pone.0156575.s004.doc]

S1 Table. Full-text excluded articles with reasons for exclusion (n=38).

| Accession Number | Year | Jounal | Title | Author | Result | Reason |
| --- | --- | --- | --- | --- | --- | --- |
| 25134942 | 2014 | Int Urol Nephrol | Relationship between prehypertension and incidence of chronic kidney disease in a general population: a prospective analysis in central south China | X. Cao, X. Xie, J. Zhou, H. Yuan and Z. Chen | excluded | Not report RR/95%CI |
| 26744126 | 2015 | Am J Kidney Dis | Prediabetes and Risk of Glomerular Hyperfiltration and Albuminuria in the General Nondiabetic Population: A Prospective Cohort Study | T. Melsom, J. Schei, V. T. Stefansson, M. D. Solbu, T. G. Jenssen, U. D. Mathisen, T. Wilsgaard and B. O. Eriksen | excluded | Not report RR/95%CI |
| 18400822 | 2008 | NDT | Predictors of change in estimated GFR: a population-based 7-year follow-up from the Tromso study | J. KronborgM. SolbuI. NjolstadI. ToftB. O. EriksenT. Jenssen | excluded | Not report RR/95%CI |
|  | 2014 | Transplantation | Risk-assessment for potential live-kidney donors: Predicting egfr <60 ml/min/1.73m2 at one year after donation | V. Wadhera, D. Lapointe-Rudow, A. Weinberg, M. Sharma, Z. Ebcioglu, V. Delaney, V. Sehgal, V. Nair, S. Florman and J. Rocca | excluded | Not PreHTN |
| 14507604 | 2003 | Am J Epidemiol | Correlates of urinary albumin excretion in young adult blacks and whites: the Coronary Artery Risk Development in Young Adults Study | M. A. MurtaughDR Jr JacobsX. YuM. D. GrossM. Steffes | excluded | Not PreHTN |
| 15897369 | 2005 | Hypertension | Long-term impact of systolic blood pressure and glycemia on the development of microalbuminuria in essential hypertension | J. M. PascualE. RodillaC. GonzalezS. Perez-HoyosJ. Redon | excluded | Not PreHTN |
| 16448897 | 2006 | Am J Hypertens | Albumin-to-creatinine ratio predicts change in ambulatory blood pressure in normotensive persons: a 7.5-year prospective study | L. M. Gerber, J. E. Schwartz and T. G. Pickering | excluded | Not PreHTN |
| 16636209 | 2006 | Arch Intern Med | Association of impaired diurnal blood pressure variation with a subsequent decline in glomerular filtration rate | M. B. Davidson, J. K. Hix, D. G. Vidt and D. J. Brotman | excluded | Not PreHTN |
| 19100091 | 2008 |  | Association between metabolic syndrome and chronic kidney disease |  | excluded | Not PreHTN |
| 20054026 | 2010 | Nephrol Dial Transplant | Changes in renal risk factors versus renal function outcome during follow-up in a population-based cohort study | N. Halbesma, D. F. Jansen, R. P. Stolk, P. E. De Jong and R. T. Gansevoort | excluded | Not PreHTN |
| 22058177 | 2012 | Nephrol Dial Transplant | A study of the natural history of diabetic kidney disease (DKD) | N. Altemtam, J. Russell and N. M. El | excluded | Not PreHTN |
| 23340164 | 2013 | J Hypertens | High-normal diastolic blood pressure is a risk for development of microalbuminuria in the general population: the Watari study | S. KonnoA. HozawaY. MiuraS. ItoM. Munakata | excluded | Not PreHTN |
| 23813282 | 2013 | QJM | Associations of baseline characteristics with evolution of eGFR in a referred chronic kidney disease cohort | R. A. Hoefield, P. A. Kalra, B. Lane, D. J. O'Donoghue, R. N. Foley and R. J. Middleton | excluded | Not PreHTN |
|  | 2012 |  | Importance of blood pressure control in the high risk population of chronic kidney disease: From the results of the kidney early evaluation program in Japan |  | excluded | Not PreHTN |
| 14970063 | 2004 | JAMA | Predictors of new-onset kidney disease in a community-based population | C. S. Fox, M. G. Larson, E. P. Leip, B. Culleton, P. W. Wilson and D. Levy | excluded | Not PreHTN |
| 17728802 | 2008 | J Hum Hypertens | Risk of chronic kidney disease in hypertensive patients with other metabolic conditions | D. Weycker, G. A. Nichols, M. O'Keeffe-Rosetti, J. Edelsberg, G. Vincze, Z. M. Khan and G. Oster | excluded | Not PreHTN |
| 19064831 | 2008 | Arch Intern Med | A simple algorithm to predict incident kidney disease | A. V. Kshirsagar, H. Bang, A. S. Bomback, S. Vupputuri, D. A. Shoham, L. M. Kern, P. J. Klemmer, M. Mazumdar and P. A. August | excluded | Not PreHTN |
| 20800153 | 2010 | Am J Med | A prediction model for the risk of incident chronic kidney disease | K. L. Chien, H. J. Lin, B. C. Lee, H. C. Hsu, Y. T. Lee and M. F. Chen | excluded | Not PreHTN |
| 21430373 | 2011 | Am J Nephrol | Uric acid levels predict future development of chronic kidney disease | H. Sonoda, H. Takase, Y. Dohi and G. Kimura | excluded | Not PreHTN |
| 21576832 | 2011 | Intern Med | The development of chronic kidney disease in Japanese patients with non-alcoholic fatty liver disease | Y. Arase, F. Suzuki, M. Kobayashi, Y. Suzuki, Y. Kawamura, N. Matsumoto, N. Akuta, M. Kobayashi, H. Sezaki, S. Saito, T. Hosaka, K. Ikeda, H. Kumada, Y. Ohmoto, K. Amakawa, H. Tsuji, S. D. Hsieh, K. Kato, M. Tanabe, K. Ogawa, S. Hara and T. Kobayashi | excluded | Not PreHTN |
| 21722384 | 2011 | BMC Nephrol | Association between asymptomatic hyperuricemia and new-onset chronic kidney disease in Japanese male workers: a long-term retrospective cohort study | M. Kawashima, K. Wada, H. Ohta, H. Terawaki and Y. Aizawa | excluded | Not PreHTN |
| 23772149 | 2013 | J Korean Med Sci | The association between uric acid and chronic kidney disease in Korean men: a 4-year follow-up study | J. H. Ryoo, J. M. Choi, C. M. Oh and M. G. Kim | excluded | Not PreHTN |
| 24434843 | 2014 | Nephron Clin Pract | Hyperuricemia is a significant risk factor for the onset of chronic kidney disease | A. Toda, Y. Ishizaka, M. Tani and M. Yamakado | excluded | Not PreHTN |
| 24970884 | 2014 | J Am Soc Nephrol | Mid-adulthood risk factor profiles for CKD | G. M. McMahon, S. R. Preis, S. J. Hwang and C. S. Fox | excluded | Not PreHTN |
| 25673040 | 2015 | Am J Hypertens | Blood Pressure, Proteinuria, and Renal Function Decline: Associations in a Large Community-Based Population | A. Hirayama, T. Konta, K. Kamei, K. Suzuki, K. Ichikawa, S. Fujimoto, K. Iseki, T. Moriyama, K. Yamagata, K. Tsuruya, K. Kimura, I. Narita, M. Kondo, K. Asahi, I. Kurahashi, Y. Ohashi and T. Watanabe | excluded | Not PreHTN |
| 17984108 | 2008 | Nephrol Dial Transplant | Blood pressure measures and risk of chronic kidney disease in men | E. S. SchaeffnerT. KurthT. S. BowmanR. P. GelberJ. M. Gaziano | excluded | Not compared PreHTN with NBP |
| 22183043 | 2012 | Blood Press Monit | Association of home blood pressure variability with progression of chronic kidney disease | T. Okada, H. Matsumoto, Y. Nagaoka and T. Nakao | excluded | Not compared PreHTN with NBP |
|  | 2012 | Hypertension | Renal dysfunction associated with incident hypertension according to blood pressure categories in a non-hypertensive population in the suita study: An urban cohort study | Y. Kokubo, M. Watanabe, S. Nakamura, K. Kawanishi and Y. Miyamoto | excluded | Not compared PreHTN with NBP |
|  | 2015 | Circulation | Blood pressure during pregnancy and risk of hypertension later in life: A longitudinal study of pouchmoms | G. L. Dunietz, K. L. Strutz, C. B. Holzman, Y. Tian, D. Todem, B. L. Bullen and J. M. Catov | excluded | Not CKD |
| 12860578 | 2003 | Arch Intern Med | Effects of blood pressure level on progression of diabetic nephropathy: results from the RENAAL study | G. L. Bakris, M. R. Weir, S. Shanifar, Z. Zhang, J. Douglas, D. J. van Dijk and B. M. Brenner | excluded | Not CKD |
| 16421161 | 2006 | Nephrol Dial Transplant | Association of low blood pressure with increased mortality in patients with moderate to severe chronic kidney disease | C. P. Kovesdy, B. K. Trivedi, K. Kalantar-Zadeh and J. E. Anderson | excluded | Not CKD |
| 19339424 | 2009 | Clin J Am Soc Nephrol | Blood pressure components and the risk for end-stage renal disease and death in chronic kidney disease | R. Agarwal | excluded | Not CKD |
| 7494564 | 1996 | N Engl J Med | Blood pressure and end-stage renal disease in men | M. J. KlagP. K. WheltonB. L. RandallJ. D. NeatonF. L. BrancatiC. E. FordN. B. ShulmanJ. Stamler | included | Not CKD |
| 12707291 | 2003 | Hypertension | Blood pressure predicts risk of developing end-stage renal disease in men and women | M. TozawaK. IsekiC. IsekiK. KinjoY. IkemiyaS. Takishita | included | Not CKD |
| 14569104 | 2003 | J Am Soc Nephrol | Risk factors for chronic kidney disease: a prospective study of 23,534 men and women in Washington County, Maryland | M. K. HarounB. G. JaarS. C. HoffmanG. W. ComstockM. J. KlagJ. Coresh | included | Not CKD |
| 15851645 | 2005 | Arch Intern Med | Elevated blood pressure and risk of end-stage renal disease in subjects without baseline kidney disease | C. Y. HsuC. E. McCullochJ. DarbinianA. S. GoC. Iribarren | included | Not CKD |
| 17475822 | 2007 | J Am Soc Nephrol | A population-based, prospective study of blood pressure and risk for end-stage renal disease in China | K. ReynoldsD. GuP. MuntnerJ. W. KusekJ. ChenX. WuX. DuanC. S. ChenM. J. KlagP. K. WheltonJ. He | included | Not CKD |
| 19515474 | 2009 | Am J Kidney Dis | Prehypertension, obesity, and risk of kidney disease: 20-year follow-up of the HUNT I study in Norway | J. MunkhaugenS. LydersenT. E. WideroeS. Hallan | included | Not CKD |
